# A besyian regularisation neural network approach for hepatitis B virus spread prediction and immune system therapy model

**DOI:** 10.1038/s41598-024-75336-x

**Published:** 2024-10-10

**Authors:** Ahmed M. Galal, Qusain Haider, Ali Hassan, Mubashar Arshad, Mohammad Mahtab Alam, Laila A. Al-Essa, Haile Habenom

**Affiliations:** 1 Department of Mechanical Engineering, College of Engineering in Wadi Alddawasir, Prince Sattam bin Abdulaziz University, Wadi Alddawasir, Saudi Arabia; 2 Production Engineering and Mechanical Design Department, Faculty of Engineering, Mansoura University, P.O 35516, Mansoura, Egypt; 3 Department of Mathematics, University of Gujrat, Gujrat, 50700 Pakistan; 4 Department of Mechanics and Aerospace Engineering, Southern University of Science and Technology, Shenzhen, Guangdong China; 5 Department of Mathematics, Abbottabad University of Science & Technology, Abbottabad, 22500 Pakistan; 6 Department of Basic Medical Sciences, College of Applied Medical Science, King Khalid University, Abha, 61421 Saudi Arabia; 7 Department of Mathematical Sciences, College of Science, Princess Nourah bint Abdulrahman University, P.O.Box 84428, Riyadh, 11671 Saudi Arabia; 8 Department of Mathematics, Wollega University, Nekemte, Ethiopia

**Keywords:** Hepatitis B virus, Immune system, Bayesian regularization method, Neural network, Numerical solutions, Infectious diseases, Medical research

## Abstract

The primary aim of the article is to analyze the response of the human immune system when it encounters the hepatitis B virus. This is done using a mathematical system of differential equations. The differential equation system has six components, likely representing various aspects of the immune response or virus dynamics. A Bayesian regularization neural network has been presented in the process of training. These networks are employed to find solutions for different categories or scenarios related to hepatitis B infection. The Adams method is used to generate reference data sets. The back-propagated artificial neural network, based on Bayesian regularization, is trained and validated using the generated data. The data is divided into three sets: 90% for training and 5% each for testing and validation. The correctness and effectiveness of the proposed neural network model have been assessed using various evaluation metrics. The metrics have been used in this study are Mean Square Error (MSE), histogram errors, and regression plots. These measures provide support to the neural network to approximate the immune response to the hepatitis B virus.

## Introduction

The immune system serves as the body’s defense mechanism against infectious diseases, comprising a complex network of cells, tissues, and organs. Without it, the human body would be vulnerable to attacks from bacteria, viruses, and parasites. An intact immune system can discern between healthy and undesirable tissues. Researchers have explored various aspects of immune responses in different contexts.

Chener et al.^1^ discussed a mathematical model detailing the immune system’s reaction to hepatitis B infection, while Galal et. al.^2^ introduced/explored a mathematical framework for understanding the immune response to tumor invasions. Eftimie et al.^3^ examined the interaction between the immune system and cancer using a mathematical model. Su et al.^4^ investigated immune responses in tissues and organs, and Marchuk et al.^5^ developed a mathematical model to study immune reactions in the presence of infectious diseases. Ucar et al.^6^ used a fractional-order mathematical model to describe immune cells’ behavior under the influence of cancer. Mayer and Zaenker^7^ explained the fundamentals of mathematical modeling in immune responses, while Delitala and Lorenzi^8^ explored recognition and learning aspects in a mathematical model for immune responses to cancer. Volinsky et al.^9^ provided insights into a mathematical model for hepatitis B virus treatment involving the immune system. He and Ain^10^ conducted a comprehensive analysis of future challenges in fractal calculus, considering two-scale thermodynamics and fractal variational principles. Perelson et al.^11^ focused on modeling viral immune system dynamics, and Gutnikov and Melnikov^12^ presented a simplified model for immune responses.

Mathematical modeling has long been instrumental in studying epidemics, including recent applications involving infectious diseases and fractional calculus. Keeling and Danon^13^ introduced mathematical models for understanding infectious diseases, while Huppert and Katriel^14^ discussed prediction and modeling methods, including the prediction of rapid disease spread. The COVID-19 model based on the susceptible, infected, treatment, and recovered model has been presented by Sánchez et al.^15^, and singular two-point boundary value problems arising in the theory of thermal explosion have been proposed by Sabir et al.^16^, mosquito dispersal model in a heterogeneous environment has been provided by Umar et al.^17^, modeling of heat conduction in the human head is presented by Raja et al.^18^, a novel third-order nonlinear Emden–Fowler delay differential model has been presented by Guirao et al.^19^, Gudermannian neural network for solving the SITR fractal system has been derived by Sabir et al.^20^, and COVID-19 model including government strategies and individual responses has been proposed by Botmart et al.^21^.

Yusuf et al.^22^ conducted a study on pine wilt disease using the Caputo fractional operator. Baba et al.^23^ examined the restrictions imposed during the COVID-19 lockdowns through a mathematical model. Ain et al.^24^ explored the optimal variational iteration method for addressing parametric boundary value problems. Er et al.^25^ introduced a diagnostic mechanism for tuberculosis employing an artificial neural network. Agrebi and Larbi^26^ delved into the application of artificial intelligence in the study of infectious diseases. Sabir et al.^27^ addressed infectious disease modeling by incorporating anatomical variables and stochastic procedures. Tran et al.^28^ discussed the evolving applications of artificial neural networks and machine learning in modeling infectious diseases. Allugunti et al.^29^ investigated skin disease using a machine-learning model based on convolutional neural networks. Aslan et al.^30^ outlined a methodology for diagnosing COVID-19 using CNN and Bayesian optimization. Kumar et al.^31^ presented a fractional-order mathematical model of COVID-19, considering the presence of vaccination. Shawaqfah and Almomani^32^ provided an analysis of the COVID-19 outbreak in Qatar, Italy, and Spain using an artificial neural network. Wong et al.^33^ employed an artificial neural network to analyze infectious diseases. Shaoib et al.^34^ conducted stochastic numerical computations to examine the dynamics of COVID-19 using an artificial neural network with a nonlinear SITR system.

This research demonstrates the effectiveness of the Levenberg-Marquardt Backpropagation (LMB) method combined with artificial neural networks (ANNs) in modeling the differential system of the hepatitis B virus and its interaction with the immune system. This novel approach has not been previously applied to the hepatitis B virus model with immune system treatment using integer-order derivatives. The study employs computational stochastic methods to address complex dynamics. It distinguishes itself from other studies, such as food chain modeling^35^, COVID-19 dynamics^36^, and nonlinear disease models^37–40^ by focusing on the hepatitis B virus model.

Key features of this study include:

- A unique numerical solution and differential equations for the hepatitis B virus model with immune system therapy.
- Numerical simulations of the hepatitis B virus model with the immune system using stochastic techniques with the ANNs-BRM solver.
- Presentation of three different dissimilarity measures for the hepatitis B virus model with immune system treatment, numerically computed with the ANNs-BRM solver.
- Assessment of the accuracy of the ANNs-BRM solver through absolute error (AE) values, demonstrating its reliability.
- Evaluation of the ANNs-BRM solver’s performance using regression (REG) values, standard errors (STs), error histograms (EHs), mean square error (MSE), and correlation measures for solving the hepatitis B virus model with immune system interactions.

The paper is structured as follows: Sect. 2 presents the mathematical form of the hepatitis B virus differential system. Section 3 outlines the research methodology, while Sect. 4  provides the results and their analysis. The paper concludes with final remarks in the last section.

## Mathematical formulations of the model

Recently, Volinsky and colleagues^9^ introduced a mathematical model to describe the dynamics of the Hepatitis B virus when treated with immune system therapy. This mathematical model is built upon a system of six differential equations, each representing a specific variable that characterizes the relationship between the virus and the host over time. These variables are denoted as X(t), Y(t), V(t), W(t), Z(t), and G(t), and they correspond to uninfected cells, infected cells, free virus particles, antibody response, cytotoxic T lymphocyte (CTL) response, and the treatment’s effect on the immune system, respectively. Uninfected cells (X) replicate at a rate of r, experience natural cell death at a rate of dX, and can become infected by the virus at a rate of bVX. Cytotoxic T lymphocytes (CTLs) are responsible for targeting infected cells (Y) and have a growth rate determined by bVX, a natural death rate of aY, and an ability to kill infected cells at a rate of pXZ. The antibody response (W) develops in reaction to the presence of free virus particles (V) at a rate of gVW and degrades at a rate of hW. The number of CTLs (Z) increases due to the presence of infected cells (Y) at a rate of cYZ and decreases in the absence of infection at a rate of bZ. Free virus particles (V) are produced by infected cells (Y) at a rate of kY, degrade at a rate of uV, and can be neutralized by antibodies (W) at a rate of qVW. Overall, this mathematical model provides a framework for understanding how these variables interact and influence the dynamics of Hepatitis B virus infection and its response to immune system therapy. The set of non-linear equations listed below served as the model definition:

1 $$\:\left\{\begin{array}{l}\frac{dX}{dt}=r-dX\left(t\right)-(1-\eta\:)\beta\:V\left(t\right)X\left(t\right)\:X\left(0\right)={k}_{1},\\\:\frac{dY}{dt}=(1-\eta\:)\beta\:V\left(t\right)X\left(t\right)-aY\left(t\right)-pY\left(t\right)Z\left(t\right)\:Y\left(0\right)={k}_{2},\\\:\frac{dV}{dt}=(1-\epsilon\:)kY\left(t\right)-uV\left(t\right)-qV\left(t\right)W\left(t\right)\:V\left(0\right)={k}_{3},\\\:\frac{dW}{dt}=-hW\left(t\right)+gV\left(t\right)W\left(t\right)\:W\left(0\right)={k}_{4},\\\:\frac{dZ}{dt}=cY\left(t\right)Z\left(t\right)-bZ\left(t\right)+DG\left(t\right)\:Z\left(0\right)={k}_{5},\\\:\frac{dG}{dt}=Z\left(t\right)-\alpha\:G\left(t\right)\:G\left(0\right)={k}_{6}.\end{array}\right\}$$

Here $\:t$ represents the time. Table 1 shows the description of the parameters used above.

**Table 1:** Parameters and their description and estimations are given in Sect. 4.4.

Parameters	Description
$\:{k}_{1}$	Uninfected cells
$\:{k}_{2}$	Infected cells
$\:{k}_{3}$	Free virus numbers
$\:{k}_{4}$	Antibody response
$\:{k}_{5}$	Cytotoxic lymphocyte response
$\:{k}_{6}$	Treatment of the immune system
$\:t$	Time

We propose the concept of exponential stability with regulatory measures. To consider the patient’s immune system response, we employ a modified model (Model 1) that incorporates immune therapy support. The process of implementing the Bayesian Regularization Method-Artificial Neural Network (BRM-ANN) is structured in two main steps:

1. We create a BRM-ANN dataset using established numerical techniques such as Runge-Kutta, Adam numerical, or BDF solvers. We provide a comprehensive explanation of this dataset.
2. We use an approved implementation method for BRM-ANN to approximate the solution of the hepatitis B virus model in the presence of the immune system, which is described by a set of equations (Model 1).

## Proposed procedures: ANN-BRM method

The Adam method has been utilized to demonstrate the key steps in the interpretation process. We have designed a neural network with fifteen hidden neurons and employed static allocation for the hepatitis B virus model combined with the immune system, allocating 91% for training and 5% each for testing and validation. In dealing with complexities such as overfitting, rapid convergence, and underfitting, we have harnessed the power of supervised artificial intelligence through ANN procedures and Bayesian regularization. These networks have been fine-tuned through rigorous iterative testing, knowledge incorporation, careful adjustments, experience, and addressing minor discrepancies within the system.

**Fig. 1:**
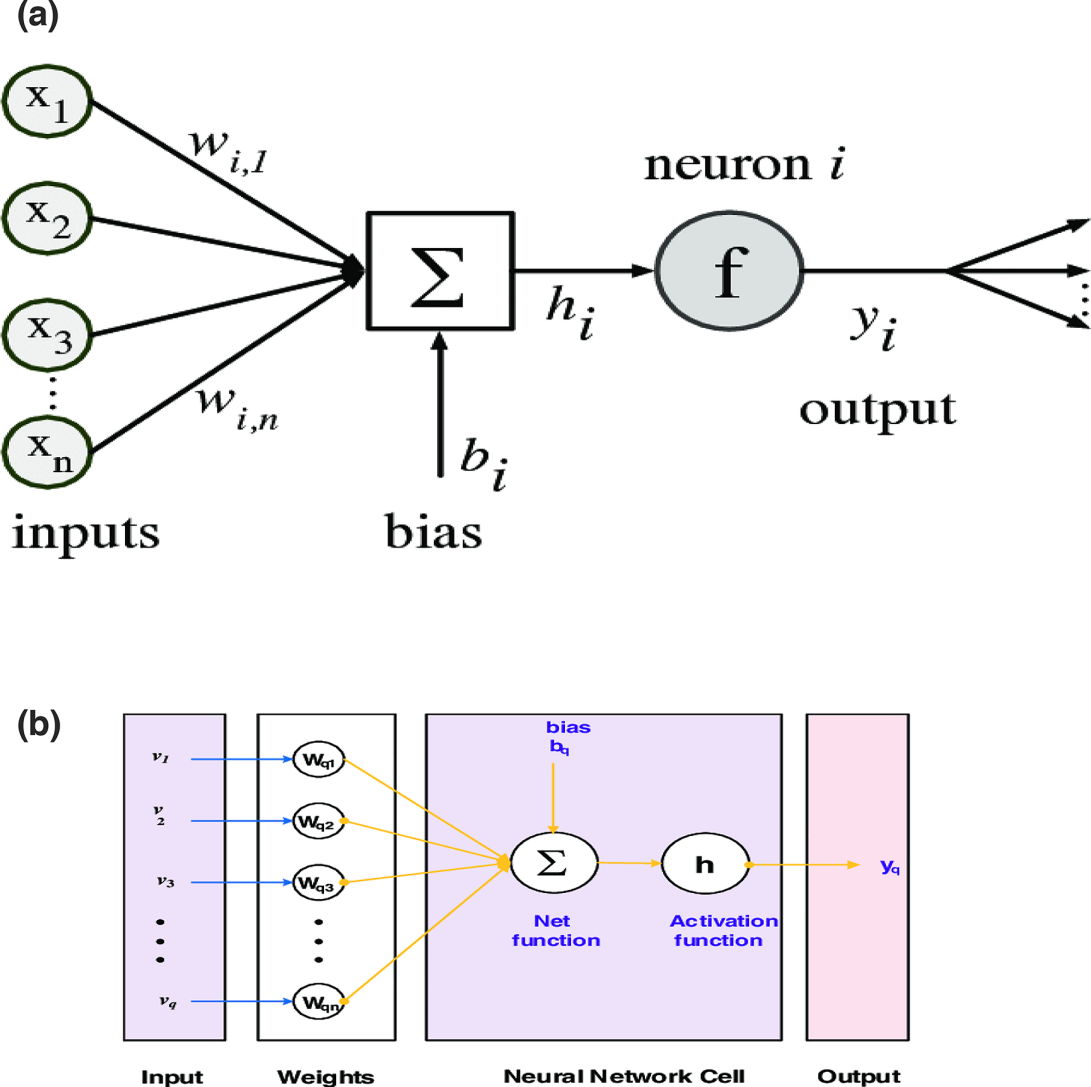
** a** Construction of single neuron, **b** Flow chart.

**Fig. 2:**
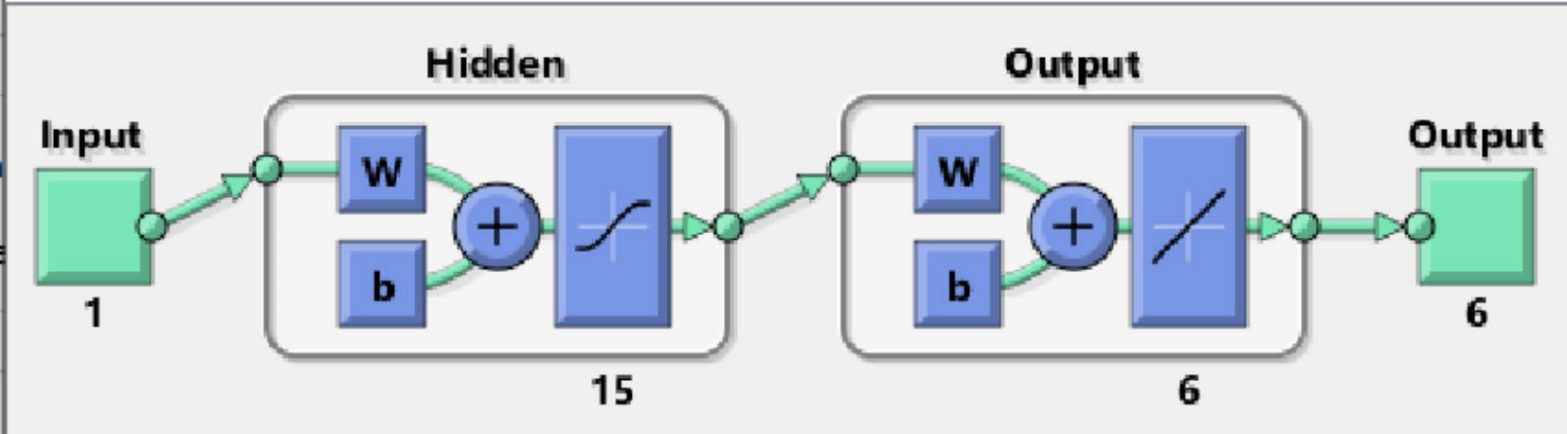
Designed hidden layer structure for the hepatitis B virus model.

The methodology is elucidated through a fundamental understanding of a single neuron, as depicted in Fig. 1(a), built upon ANN procedures and Bayesian regularization. Figure 1 illustrates the single-layered structure of the neural network, while Fig. 1(b) show a flow chart for methodology and Fig. 2 demonstrates how to configure the outer layers for solving the mathematical model of the hepatitis B virus and the immune system. The design layer consists of a single-layer input vector with fifteen neurons and six outputs. To implement ANN procedures and the Bayesian regularization method, we utilize MATLAB’s nftool command, select appropriately hidden neurons, and employ a log-sigmoid-based kernel/activation function.

The input data is obtained from numerical standard solutions, and we incorporate tolerances and step sizes for data labeling, target training, and input labeling. Default stoppage criteria are assumed. Table 2 outlines the settings and operational details of the Bayesian regularization approach for the hepatitis B virus and immune system model, including parameter configurations and the execution of ANN procedures. The Bayesian regularization method and ANN procedures are employed in tandem to facilitate system training. Fifteen numbers of neurons and log-sigmoid activation function has been used in this study.

**Table 2:** Values of the parameter to perform the stochastic scheme.

**Parameters**	**Settings **
Hidden Neurons	**15**
Fitness Measure	**0**
Maximum mu performance	**10**^**10**^
Decreasing mu performance	**0.2**
Increasing mu measure	**12**
Adaptive parameter (mu)	**7.4 × 10**^**−0.4**^
Substantiation fail amount	**6**
Maximum Epochs	**500**
Minimum gradient	**1.00 × 10**^**−07**^
Training data	**91%**
Validation data	**5%**
Testing data	** 5%**
Sample selection	***Random***
Hidden/output/input	***Single***
Datasets generation	***Adam***
Implementation and stoppage standards	***Default***

## Results and discussions

In this section, we present the distinct results obtained through the utilization of the BRM-ANN technique, and we have conducted a comprehensive analysis of these outcomes across all three cases. We have calculated mean square errors to illustrate the discrepancies between the methods, such as the Adams method employed for generating reference data and the ANN technique. Additionally, we have used histogram errors and performance plots to showcase the performance during the training, testing, and validation stages of the proposed BRM-ANN technique. Regression performance plots have also been included for each case.

Furthermore, we have validated the performance of different components within the integer-order mathematical model system using generated reference data sets for each case. We have also conducted an assessment of absolute errors for various components of the integer-order mathematical model. The following sub-sections present the outcomes for the three distinct cases, each characterized by a unique mathematical model resulting from the combination of the Bayesian regularization method and ANN procedures. These different integer-order mathematical models are outlined below.

### Case 1

Consider the dynamical immune system model with $r=6.7 \times {10^{ - 04}}$, $d=0.00333$, $\beta =07$, $a=0.56$, $p=5.14$, $k=20$, $u=0.67$, $q=5$, $h=02$, $g=05$, $c=5.14$, $b=0.112$, $\alpha =20$, $\eta =0.99$, $\varepsilon =0.5$, $D=5.5$, ${k_6}=0.6$, ${k_1}=0.1$, ${k_2}=0.1$, ${k_3}=0.3$ and ${k_4}=0.4$, ${k_5}=0.5.$

2 $$\left\{\begin{array}{l} \frac{{dX}}{{dt}}=6.7 \times {10^{ - 04}} - 0.00333X(t) - (1 - 0.99)7 V(t)X(t) \quad\quad X(0)=0.1, \hfill \\ \frac{{dY}}{{dt}}=(1 - 0.99)7 V(t)X(t) - 0.56Y(t) - 5.14Y(t)Z(t) \quad\quad\quad Y(0)=0.2, \hfill \\ \frac{{dV}}{{dt}}=(1 - 0.5)20Y(t) - 0.67 V(t) - 5 V(t)W(t) \quad\quad\quad\quad\quad\quad V(0)=0.3, \hfill \\ \frac{{dW}}{{dt}}= - 2 W(t)+5 V(t)W(t) \quad\quad\quad\quad\quad\quad\quad\quad\quad\quad\quad\quad\quad\quad W(0)=0.4, \hfill \\ \frac{{dZ}}{{dt}}=5.14Y(t)Z(t) - 0.112Z(t)+5.5G(t) \quad\quad\quad\quad\quad\quad\quad\quad\; Z(0)=0.5, \hfill \\ \frac{{dG}}{{dt}}=Z(t) - 20G(t) \quad\quad\quad\quad\quad\quad\quad\quad\quad\quad\quad\quad\quad\quad\quad\quad\quad\;\; G(0)=0.6. \hfill \\ \end{array} \right.$$

### Interpretations of results: case 1

**Fig. 3:**
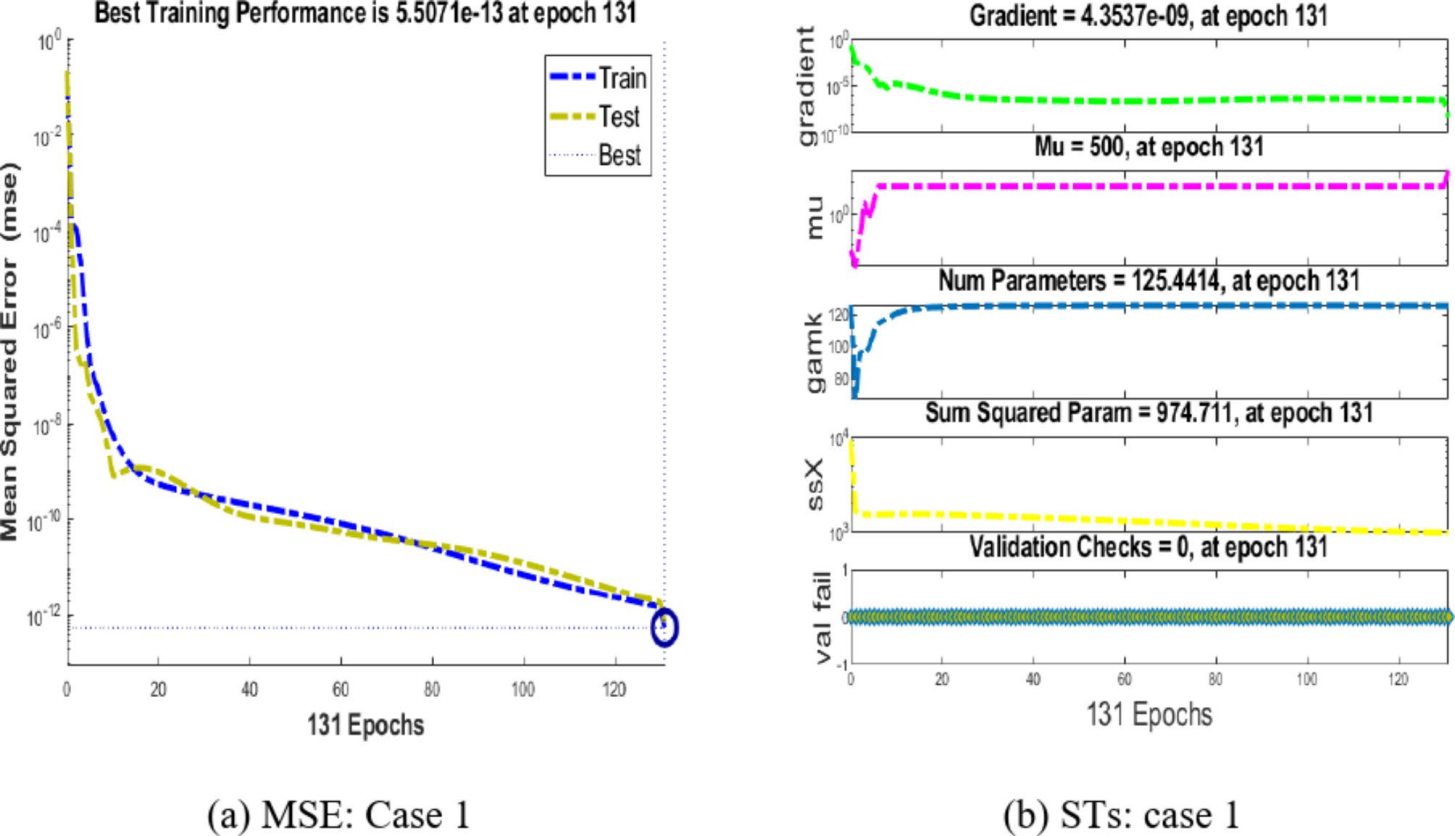
MSE and STs performances for the hepatitis model.

Figure 3(a) and (b) depict the outcomes concerning the performance of the integer-order model for the Hepatitis B virus. In Fig. 3(a), we analyze the convergence by examining the Mean Square Error (MSE) during training, testing, and validation for case 1. It’s noteworthy that the Hepatitis B virus model reaches convergence at an impressively low MSE of 10^(-12) for case 1.

Figure 3(b) illustrates the gradient and step size (Mu) for case 1. Specifically, the gradient obtained for case 1 is observed to be 4.35 × 10^(-19), and the step size is 100.5.

**Fig. 4:**
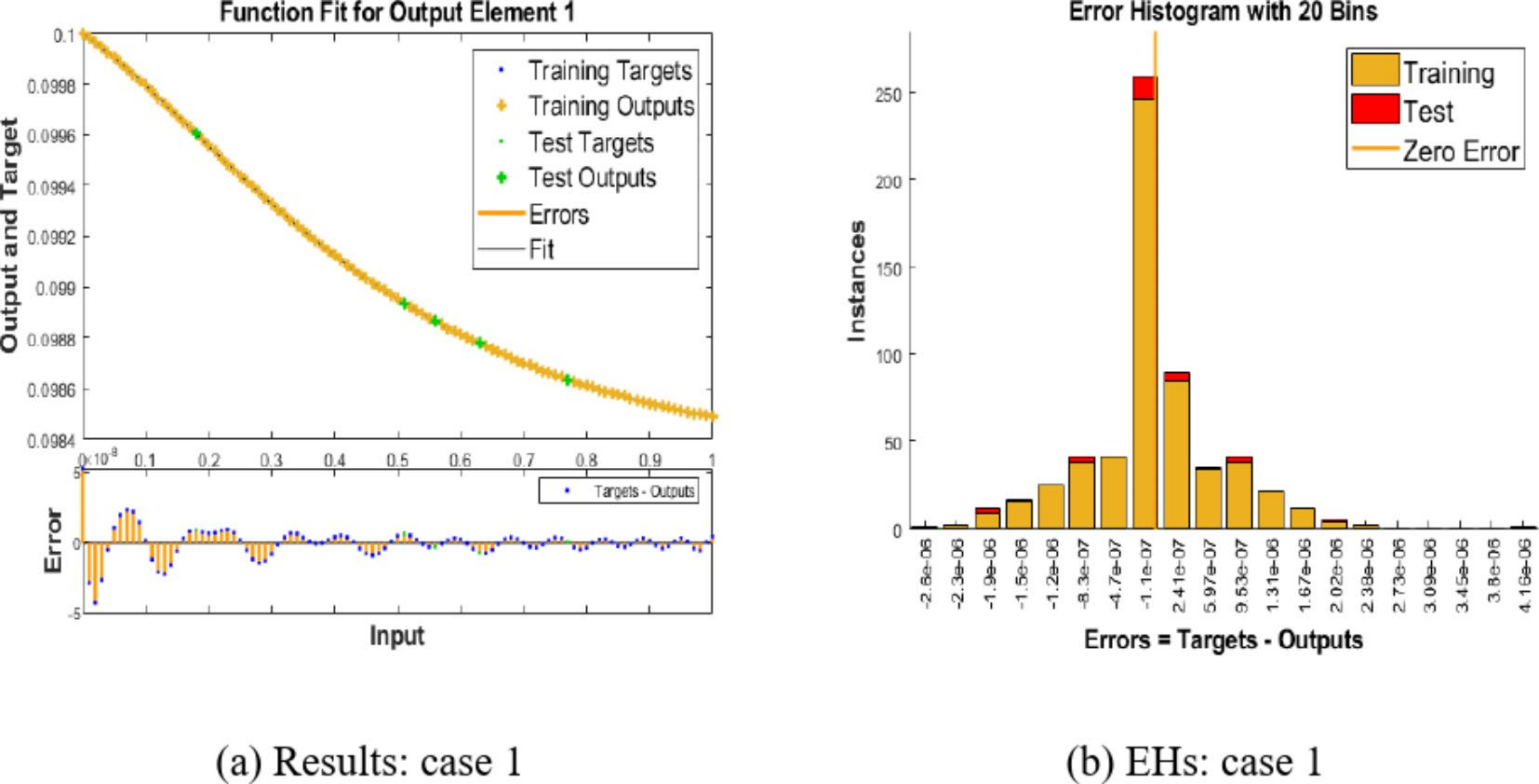
Results and EHs performances for the integer order system.

Shifting our focus to Fig. 4(a) and (b), we delve into the performance of the proposed BRM-ANN technique for the Hepatitis B virus model in case 1, along with the error histogram. In Fig. 4(a), we showcase the best performance achieved for case 1, which is an impressively low value of 5.5071 × 10^(-13). This peak performance is attained after 131 epochs. It’s worth noting that the error histograms reveal zero errors at around − 1.1 × 10^(-07), as seen in Fig. 4(b). This underscores the validation of the outcomes obtained through the proposed BRM-ANN technique.

**Fig. 5:**
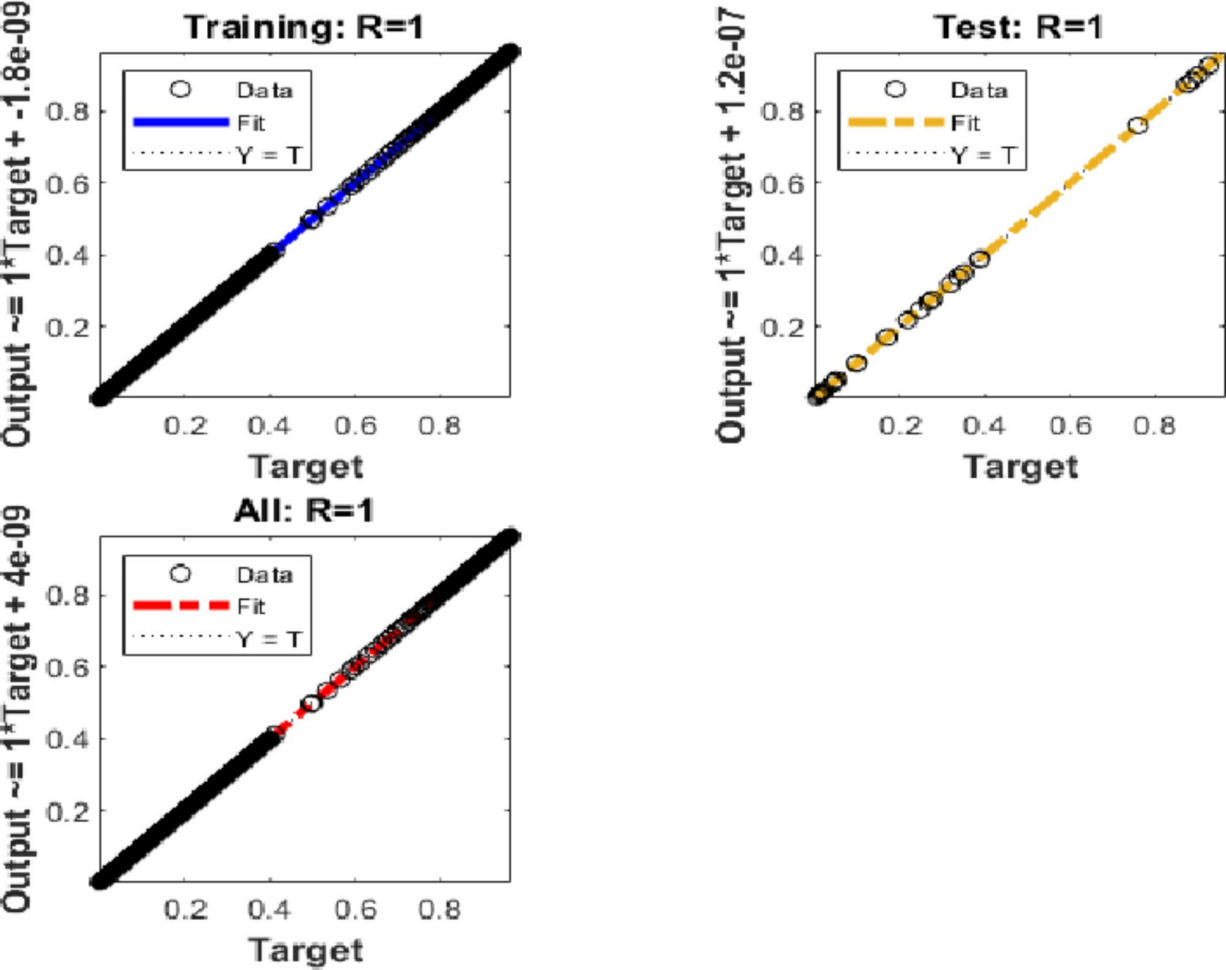
Regression performances for the integer-order system: Case 1.

Furthermore, we present a regression plot in Fig. 5 for case 1 of the Hepatitis B virus model, generated using the proposed BRM-ANN technique. This plot indicates that the correlation values (R) are all equal to unity, confirming the accuracy of the values used in the training, testing, and validation processes. Additionally, the regression plot for case 1 demonstrates the flawless performance of the integer-order mathematical model in the context of the Hepatitis B virus.

#### Case 2

Consider the dynamical immune system model with $r=6.7 \times {10^{ - 04}}$, $d=0.00333$, $\beta =07$, $a=0.56$, $p=5.14$, $k=20$, $u=0.67$, $q=5$, $h=02$, $g=05$, $c=5.14$, $b=0.112$, $\alpha =20$, $\eta =0.99$, $\varepsilon =0.5$, $D=5.5$, ${k_6}=0.5$, ${k_1}=0.1$, ${k_2}=0.1$, ${k_3}=0.3$ and ${k_4}=0.4$, ${k_5}=0.5$.

3 $$\left\{ \begin{array}{l} \frac{{dX}}{{dt}}=6.7 \times {10^{ - 04}} - 0.00333X(t) - (1 - 0.99)7 V(t)X(t) \quad\quad X=0.1, \hfill \\ \frac{{dY}}{{dt}}=(1 - 0.99)7 V(t)X(t) - 0.56Y(t) - 5.14Y(t)Z(t) \quad\quad\quad Y=0.2, \hfill \\ \frac{{dV}}{{dt}}=(1 - 0.5)20Y(t) - 0.67 V(t) - 5 V(t)W(t) \quad\quad\quad\quad\quad\quad V=0.3, \hfill \\ \frac{{dW}}{{dt}}= - 2 W(t)+5 V(t)W(t) \quad\quad\quad\quad\quad\quad\quad\quad\quad\quad\quad\quad\quad\quad W=0.4, \hfill \\ \frac{{dZ}}{{dt}}=5.14Y(t)Z(t) - 0.112Z(t)+5.5G(t) \quad\quad\quad\quad\quad\quad\quad\quad\; Z=0.5, \hfill \\ \frac{{dG}}{{dt}}=Z(t) - 20G(t) \quad\quad\quad\quad\quad\quad\quad\quad\quad\quad\quad\quad\quad\quad\quad\quad\quad\;\; G=0.5. \hfill \\ \end{array} \right.$$

### Interpretations of results: case 2

**Fig. 6:**
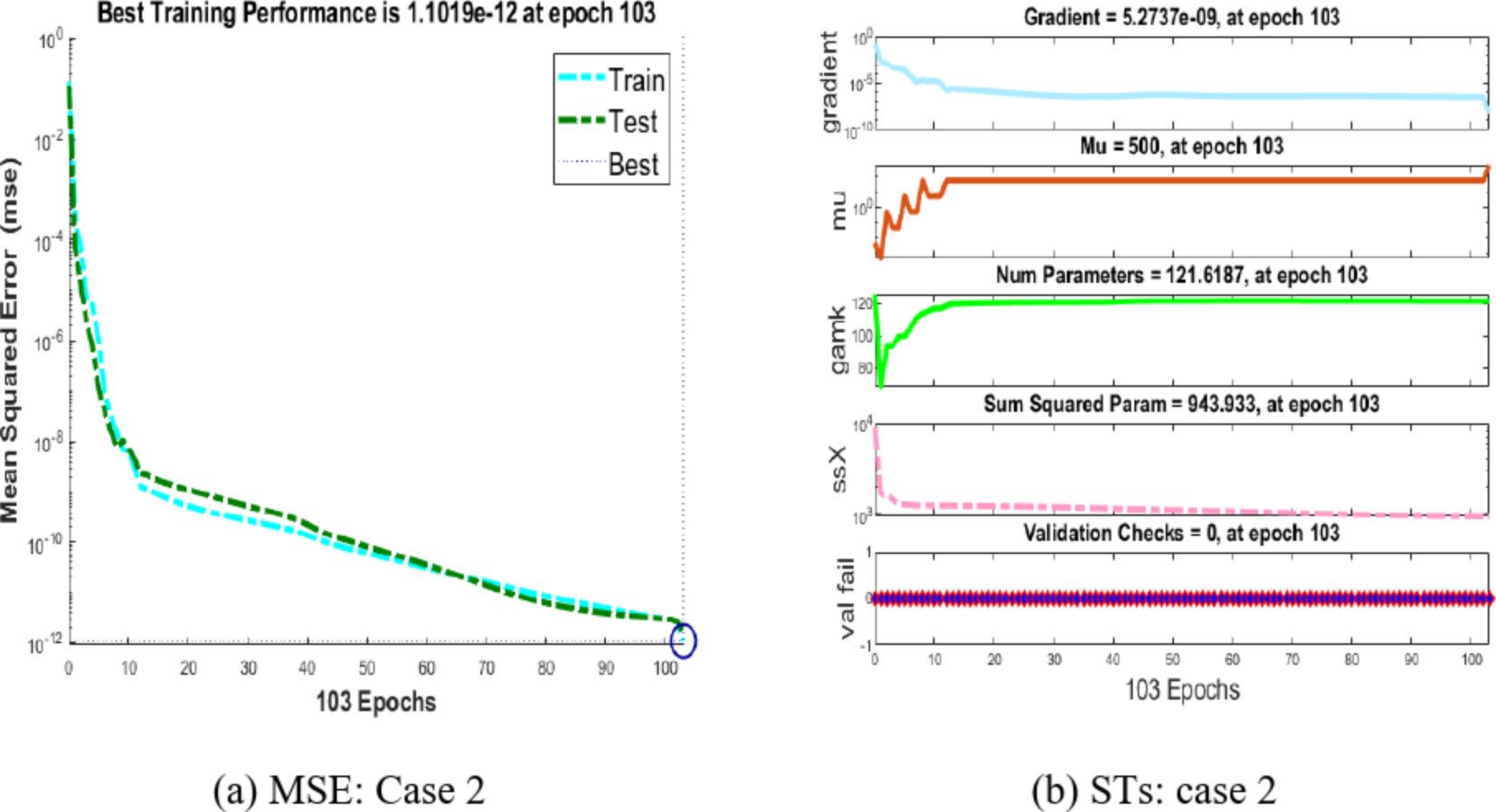
MSE and STs performances for the hepatitis model.

Figure 6(a) and (b) offer an overview of how well the integer-order Hepatitis B virus model performs. In Fig. 6(a), we analyze the convergence by assessing the Mean Square Error (MSE) during training, testing, and validation for case 2. It’s important to highlight that the Hepatitis B virus model achieves convergence at an exceptionally low MSE of 10^(-12) for case 2.

Figure 6(b) presents the gradient and step size (Mu) for case 2. Specifically, the gradient calculated for case 2 is approximately 5.2737 × 10^(-09), and the step size is 100.5.

**Fig. 7:**
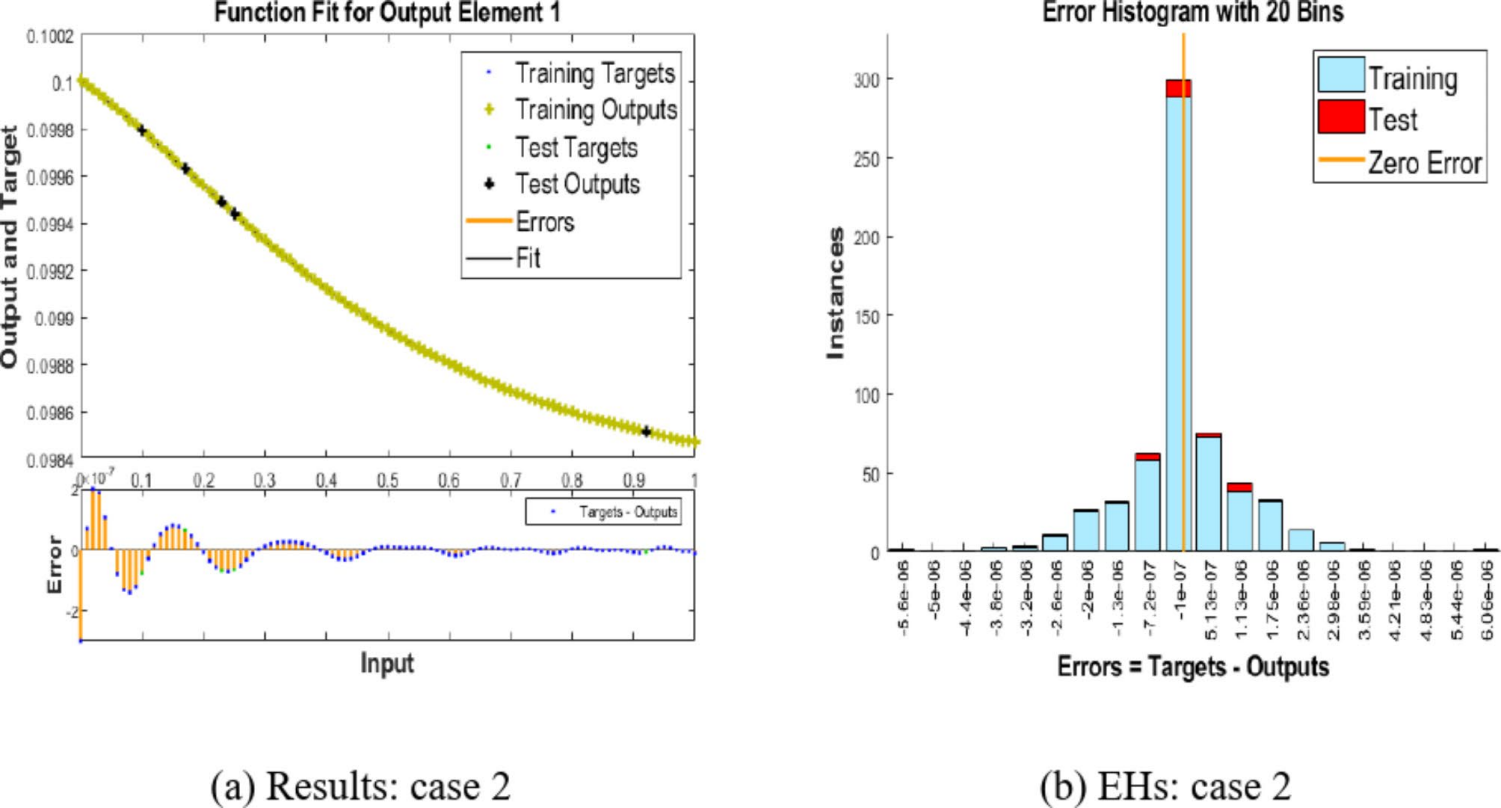
Results and EHs performances for the integer order system.

Now, let’s shift our focus to Fig. 7(a) and (b), where we explore the performance of the proposed BRM-ANN technique for the Hepatitis B virus model in case 2, along with the error histogram. In Fig. 7(a), we emphasize the peak performance achieved for case 2, which records an exceptionally low value of 2 × 10^(-07). This optimal performance is reached after 103 epochs. It’s worth noting that, as demonstrated by the error histograms, there are zero errors at around − 1 × 10^(-07), as depicted in Fig. 7(b). This serves as a confirmation of the validity of the results obtained through the proposed BRM-ANN technique.

**Fig. 8:**
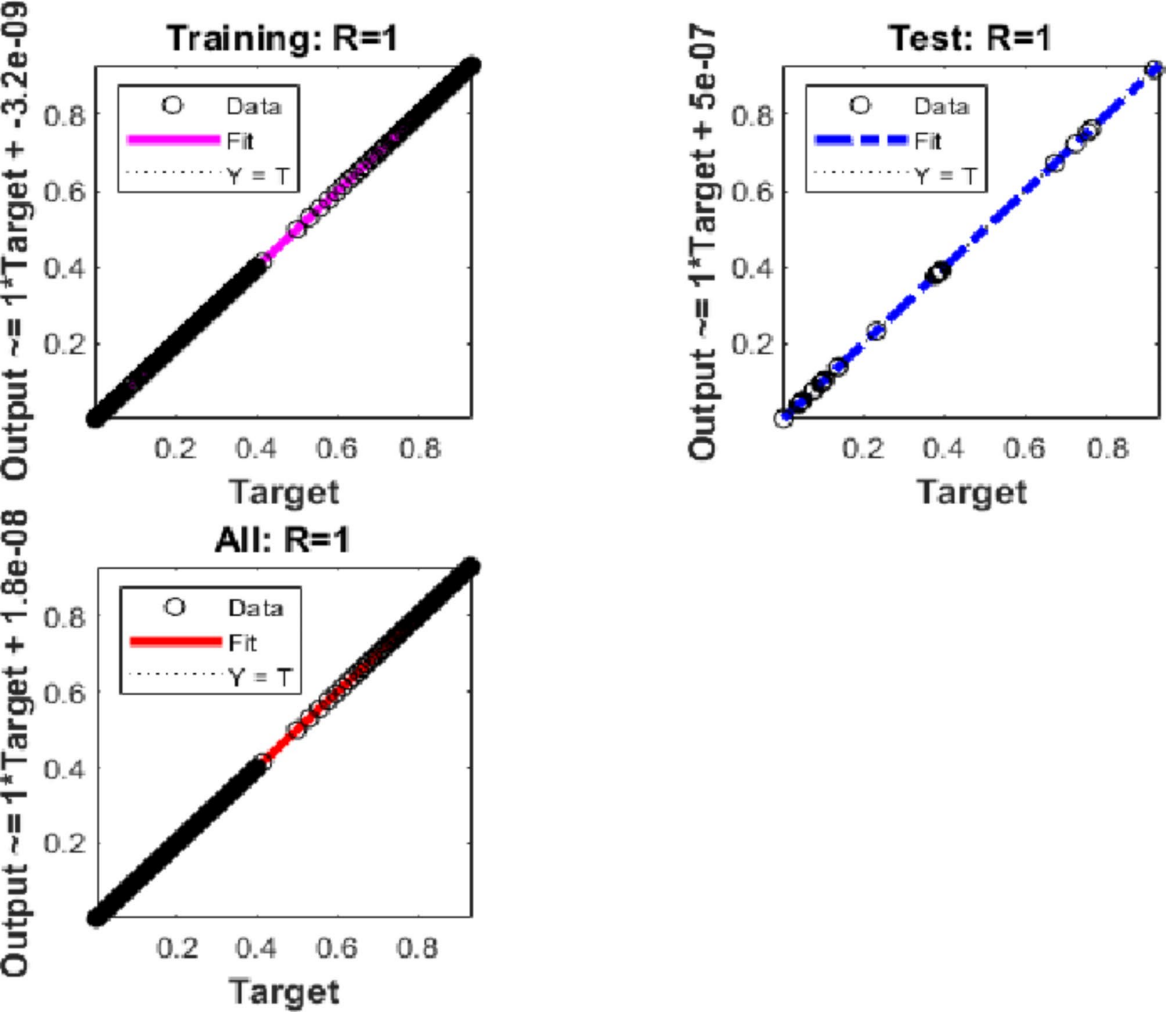
Regression performances for the integer order system: Case 2.

Furthermore, Fig. 8 presents a regression plot for case 2 of the Hepatitis B virus model, generated using the proposed BRM-ANN technique. This plot illustrates that all correlation values (R) are equal to unity, thereby confirming the accuracy of the values used in the training, testing, and validation processes. Additionally, the regression plot for case 2 showcases the flawless performance of the integer-order mathematical model in the context of the Hepatitis B virus.

#### Case 3

Consider the dynamical immune system model with $r=6.7 \times {10^{ - 04}}$, $d=0.00333$, $\beta =07$, $a=0.56$, $p=5.14$, $k=20$, $u=0.67$, $q=5$, $h=02$, $g=05$, $c=5.14$, $b=0.112$, $\alpha =20$, $\eta =0.99$, $\varepsilon =0.5$, $D=5.5$, ${k_6}=0.4$, ${k_1}=0.1$, ${k_2}=0.1$, ${k_3}=0.3$ and ${k_4}=0.4$, ${k_5}=0.5.$

4 $$\left\{ \begin{array}{l} \frac{{dX}}{{dt}}=6.7 \times {10^{ - 04}} - 0.00333X(t) - (1 - 0.99)7 V(t)X(t) \quad\quad X=0.1, \hfill \\ \frac{{dY}}{{dt}}=(1 - 0.99)7 V(t)X(t) - 0.56Y(t) - 5.14Y(t)Z(t) \quad\quad\quad Y=0.2, \hfill \\ \frac{{dV}}{{dt}}=(1 - 0.5)20Y(t) - 0.67 V(t) - 5 V(t)W(t) \quad\quad\quad\quad\quad\quad V=0.3, \hfill \\ \frac{{dW}}{{dt}}= - 2 W(t)+5 V(t)W(t) \quad\quad\quad\quad\quad\quad\quad\quad\quad\quad\quad\quad\quad\;\;\; W=0.4, \hfill \\ \frac{{dZ}}{{dt}}=5.14Y(t)Z(t) - 0.112Z(t)+5.5G(t) \quad\quad\quad\quad\quad\quad\quad\quad\; Z=0.5, \hfill \\ \frac{{dG}}{{dt}}=Z(t) - 20G(t) \quad\quad\quad\quad\quad\quad\quad\quad\quad\quad\quad\quad\quad\quad\quad\quad\quad\;\; G=0.4. \hfill \\ \end{array} \right.$$

### Interpretations of results: case 3

**Fig. 9:**
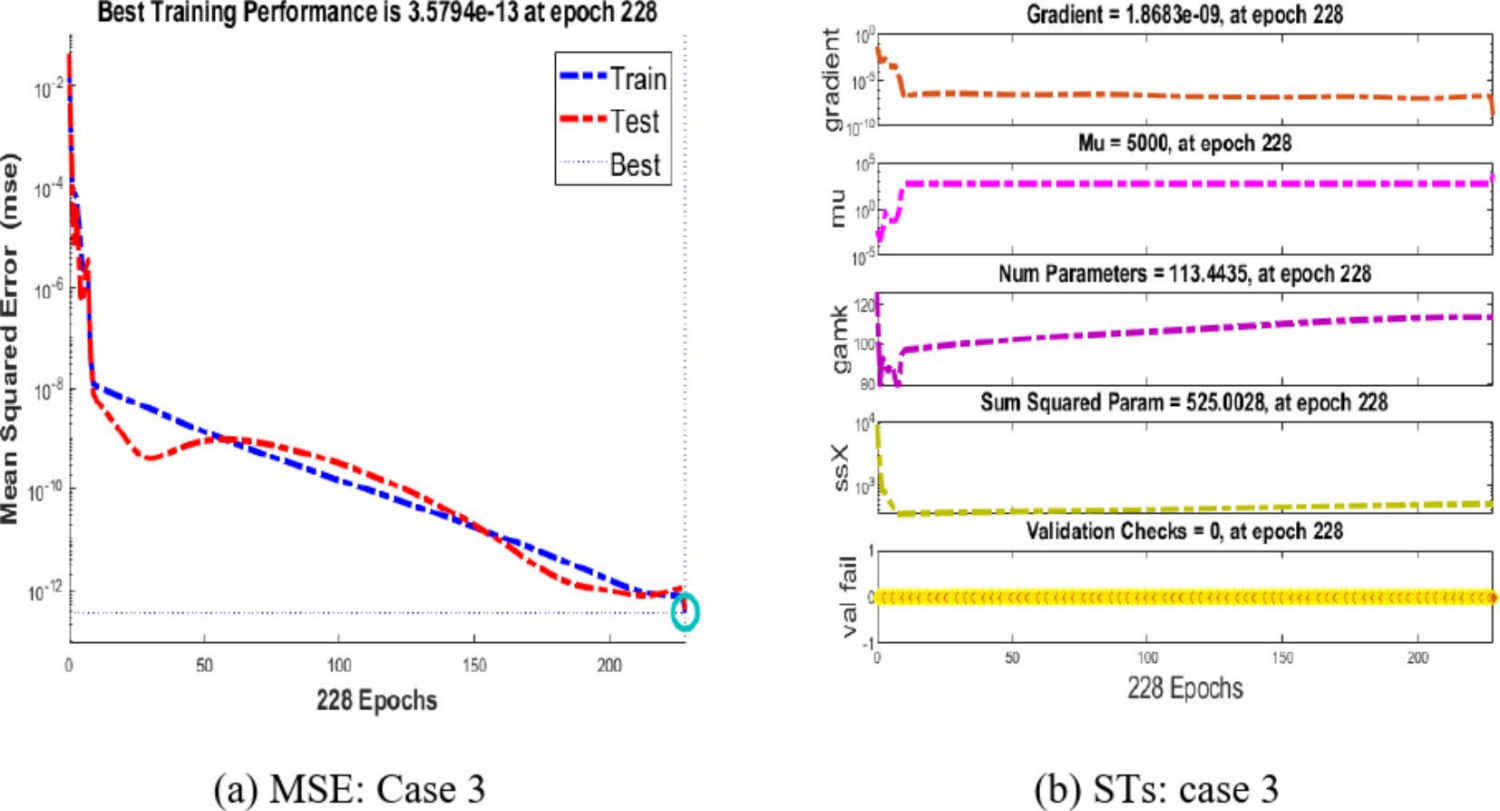
MSE and STs performances for the hepatitis model.

Integer-order calculations for case 3 are used to provide insight into the performance of the Hepatitis B virus model in Fig. 9(a) and (b). Analysing convergence in Fig. 9(a) involves looking at the Mean Square Error (MSE) during training, testing, and validation. An MSE of 10(-12) for case 3 indicates convergence of the Hepatitis B viral model.

The gradient and step size (Mu) for case 3 are shown in Fig. 9(b). For example, the step size is 105, and the estimated gradient for scenario 3 is roughly 1.868310(-09).

**Fig. 10:**
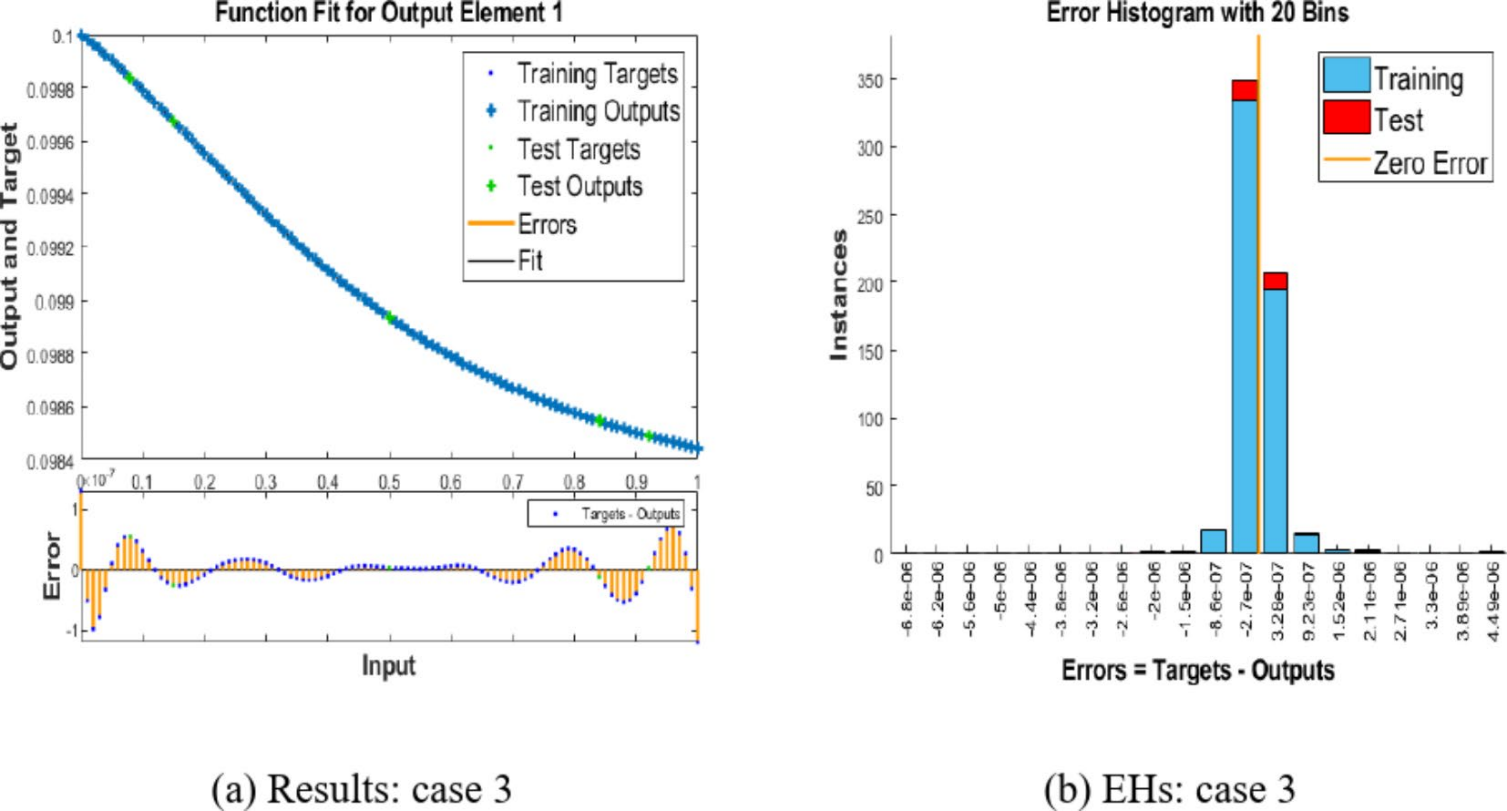
Results and EHs performances for the integer order system.

We now examine the performance of the suggested BRM-ANN technique for the Hepatitis B virus model in instance 3 along with the error histogram in Fig. 10(a) and (b). We highlight example 3’s top performance in Fig. 10(a), which records an incredibly low value of 3.579410(-13). After 103 epochs, the performance is at its peak. It is significant to notice that, as shown in Fig. 10(b), the error histograms demonstrate that there are zero errors at around − 1.110(-07). This attests to the validity of the results attained using the suggested BRM-ANN technique.

**Fig. 11:**
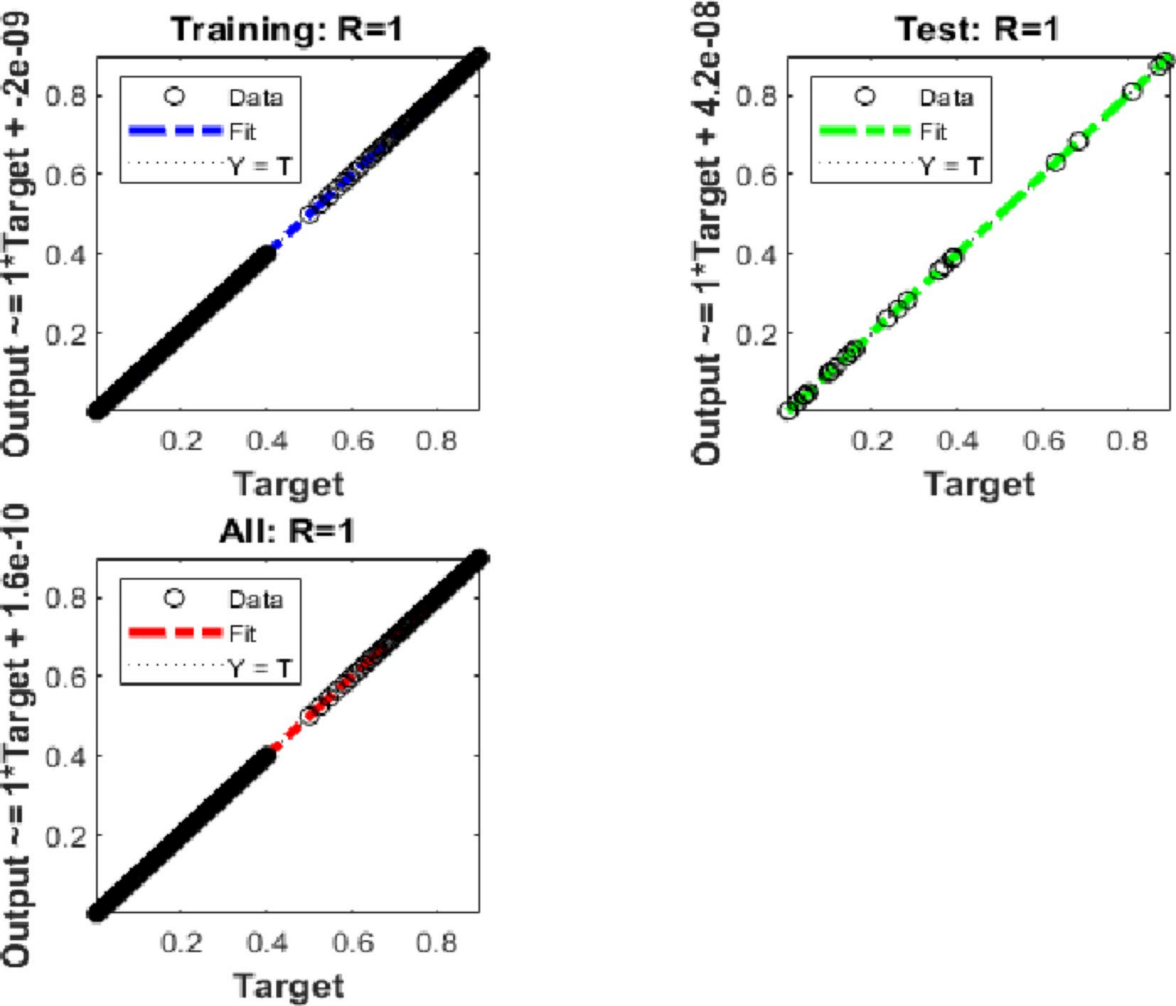
Regression performances for the integer order system.

Additionally, utilising the suggested BRM-ANN technique, Fig. 11 displays a regression curve for case 3 of the Hepatitis B virus model. The accuracy of the values used in the training, testing, and validation processes is confirmed by the correlation values (R), which are interestingly all equal to one. Additionally, the regression figure for case 3 shows the integer-order mathematical model’s perfect performance in the setting of the hepatitis B virus.

### Performance comparison of six components of HBV Therapy model

**Fig. 12:**
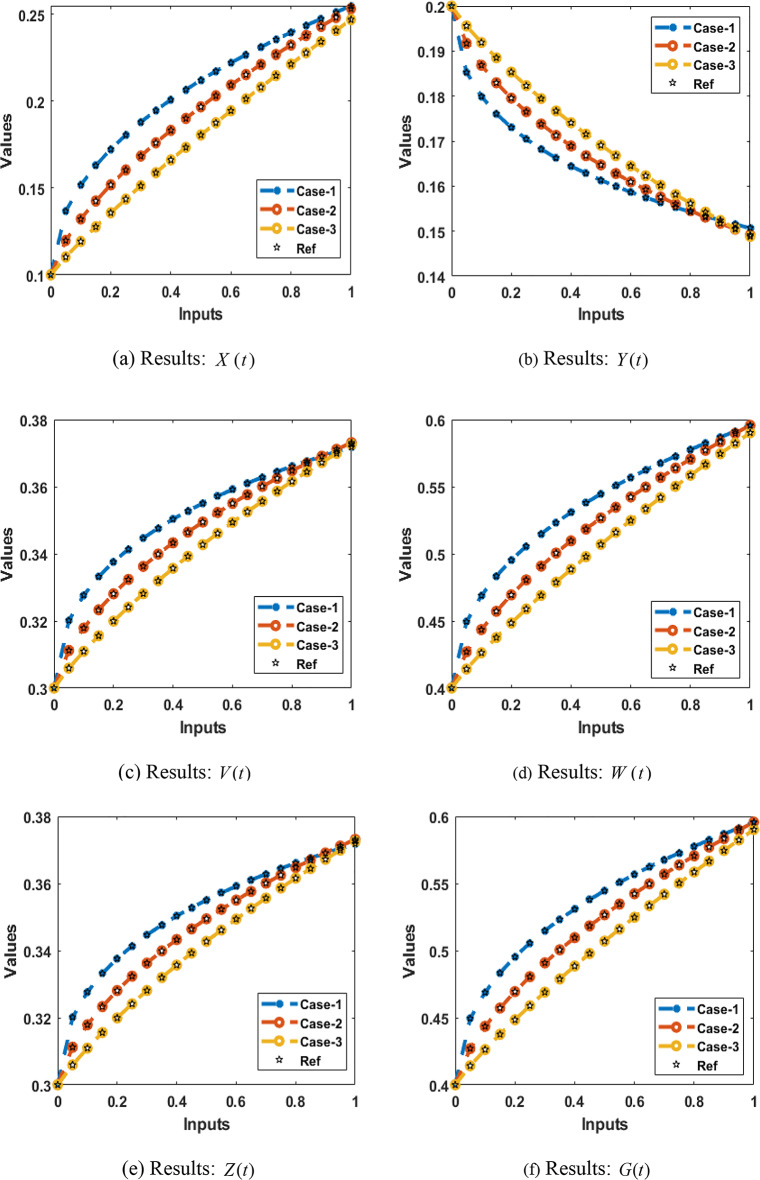
**a-f **Comparison of the performances for the integer-order system.

Figure 12(a-f) provide a thorough comparison of the results for all three cases with the reference-generated data set, taking into account factors like immune system treatment, free virus counts, antibody response, and cytotoxic T lymphocyte (CTL) response.

Figure 12(a) shows a comparison between the results of all three cases produced using the suggested BRM-ANN scheme and the uninfected cells with immune system therapy using data generated using the Adams approach. The proposed BRM-ANN scheme is effectively validated and confirmed by this comparison. In contrast to cases 2 and 3, case 1 shows a greater percentage of hepatitis B virus-free cells after immune system therapy. The poor protection for uninfected cells in the setting of the hepatitis B virus with immune system therapy is also shown in instance 3.

Figure 12(b) shows a comparison between the results obtained using the suggested BRM-ANN scheme and the generated reference data. Notably, in case 1, the amount of hepatitis B virus-positive cells rapidly decreases in the presence of immune system therapy. This comparison plot also shows that, in contrast to cases 2 and 3, case 1 causes a quicker decline in infected cells when using the hepatitis B virus model.

We compare the free viral numbers of the hepatitis B virus model with immune treatment for various instances in Fig. 12(c). The hepatitis B virus is known to produce more free viruses when there are more interactions. As an illustration, case 3 exhibits the lowest levels of hepatitis B virus-free viruses, whereas case 1 has the highest levels.

The comparison of reference data and results obtained with the suggested method for the effective response of antibodies in all three scenarios is shown in Fig. 12(d). In contrast to cases 2 and 3, which induce slower immune responses, case 1’s formulation results in a more potent antibody response for immune therapy against the hepatitis B virus model.

Figure 12(e) and (f) compare the immune system’s treatment and CTL response, respectively. Notably, when compared to case 2 and case 3, the CTL therapy successfully fights the hepatitis B virus under case 1 formulation. Comparing cases 1 and 2, it can be shown that the accuracy of the CTL response for the immune system decreases as the mathematical model is altered (See Fig. 12(e)). Figure 12(f) describes in detail how the proposed method was used to accomplish the outcomes in the three scenarios and compares them to the reference data. It is clear that treatment works better in case 1, and the accuracy and efficacy of the treatment decrease when the mathematical formulation of the hepatitis B virus model varies.

### Absolute error of six components of HBV Therapy model

**Fig. 13:**
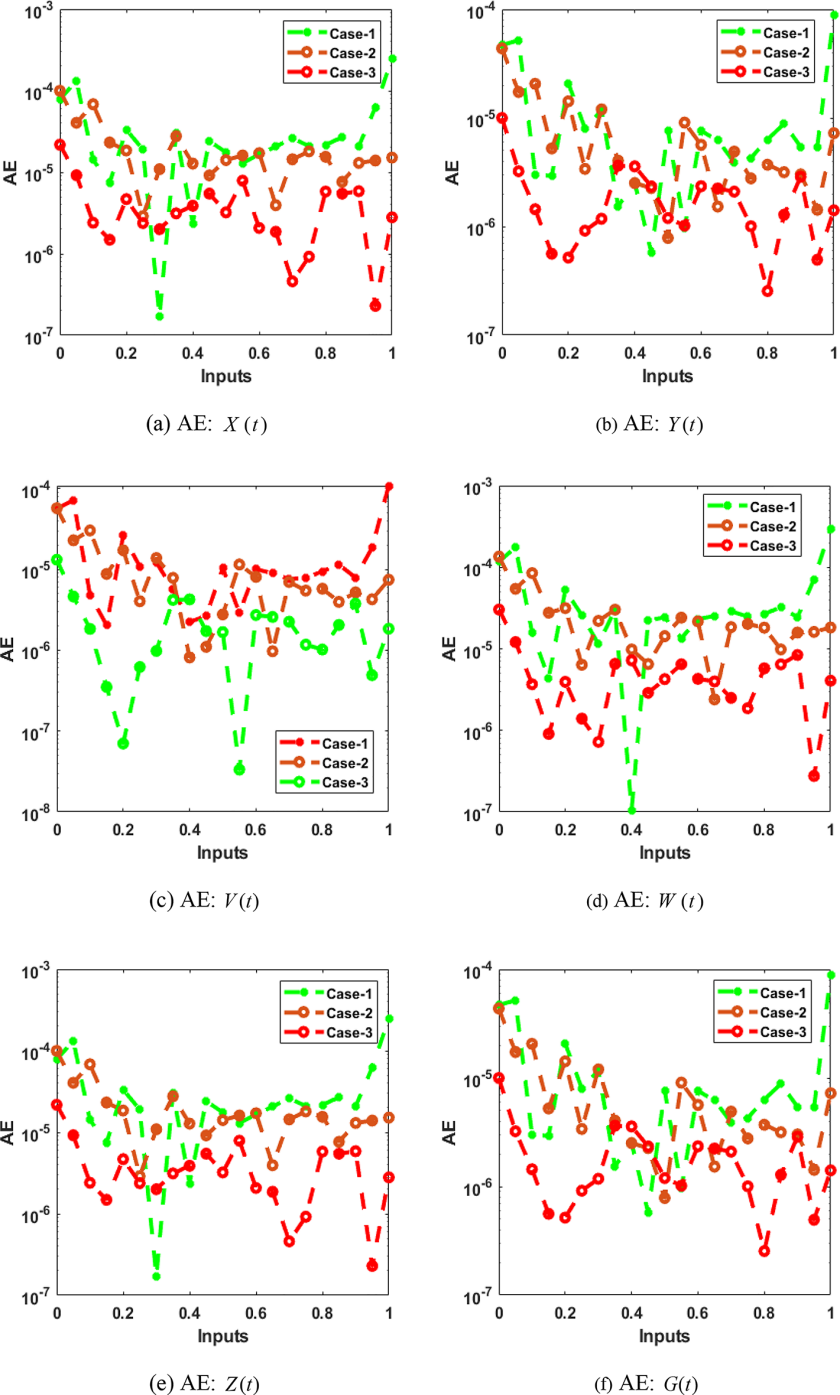
AE for the performances of the integer-order system.

The effectiveness of ANN procedures in conjunction with the Bayesian regularisation method for solving the hepatitis B virus model with the immune system is evaluated using outcome comparisons and Absolute Error (AE) measures in Fig. 13(a-f). These data demonstrate the accuracy of the ANNs process in combination with the Bayesian regularisation process for each component of the virus model, including X(t) (uninfected cells), Y(t) (infected cells), V(t) (free virus numbers), W(t) (antibody response), Z(t) (cytotoxic T lymphocyte response), and G(t) (treatment of the immune system).

For the mathematical model in all three scenarios, the AE measures for the uninfected category X(t) are shown in Fig. 13(a), ranging from 10(-04) to 10(-03). The AE values shown in Fig. 13(b) for the category of infected cells Y(t) range from 10(-05) to 10(-04) when the mathematical model is solved in each of the three scenarios.

In the three unique scenarios of the developed mathematical model of the hepatitis B virus, the free viral numbers V(t), which are measured in the range of 10(-05) to 10(-03), are shown as AE in Fig. 13(c). The AE measurements for the antibody category W(t) are also shown in Fig. 13(d), and they fall within the range of 10(-04) to 10(-03) for this model.

To solve the mathematical model, Fig. 13(e) displays AE values for the cytotoxic T cell class Z(t), ranging from 10(-04) to 10(-03). The immune system G(t) for the hepatitis B virus model with the immune system is treated with AE, assessed in the range of 10(-05) to 10(-03), in Fig. 13(f). When used in conjunction with the Bayesian regularisation approach to solve the mathematical system underlying the hepatitis B virus model, these AE representations jointly validate the correctness and precision of the ANNs procedures.

The complexity of the processes, including training performance, verification, creation, testing, and back-propagation, is characterized by the integration of Mean Square Error (MSE). These specifics are thoroughly explained in Table 3, which also shows how the hepatitis B virus model with the immune system may be addressed using ANNs processes and the Bayesian regularisation process.

**Table 3:** Bayesian regularization process to solve the hepatitis B virus with the immune model.

Case	MSE			Performance	Gradient	Mu	Epoch	Time
	[Training]	[Param]	[Testing]					
1	5.70 × 10^−13^	975	7.27 × 10^−13^	5.51 × 10^−13^	4.35 × 10^−09^	500	131	1
2	1.10 × 10^−12^	944	1.51 × 10^−12^	1.10 × 10^−12^	5.27 × 10^−09^	500	103	1
3	3.57 × 10^−13^	525	4.08 × 10^−13^	3.58 × 10^−13^	1.87 × 10^−09^	5.00 × 10^+03^	228	1

## Conclusion

This study aims to employ stochastic computing techniques to solve the integer-order hepatitis B virus model with immune system treatment using artificial neural networks and Bayesian regularization. The use of integer-order derivatives is applied to ensure precise results in modeling the hepatitis B virus with immune system treatment. The system of differential equations in this model comprises six components: X (uninfected cells), Υ (infected cells), V (free virus numbers), W (antibody response), Z (Cytotoxic T lymphocyte), and G(t) (immune system).

Key conclusions of this study include:

1. The study provides numerical results for three different variations of the hepatitis B model using ANNs and the Bayesian regularization technique.
2. The dataset for training the dynamical system consists of 91% of the data, with 5% each for testing and validation.
3. The Mean Absolute Error (AE) is calculated for each variation of the mathematical model, demonstrating the accuracy of the proposed scheme.
4. Comparison with reference solutions confirms the accuracy of the designed ANNs and the Bayesian regularization approach.
5. The Bayesian regularization method is enhanced by statistical performance evaluations of the ANNs procedure, ensuring its rationality, capability, reliability, and precision.

In the future, ANNs combined with the Bayesian regularization approach will be employed to address fractional and fluid system types.

### Advantages

Here are some of the attributes of using ANN-BRM:

1. Performance plots demonstrate that ANN-BRM is highly effective and accurate in simulating integer-order models.
2. ANN-BRM shows remarkable accuracy in predicting Mean Square Errors (MSEs) between reference data generated using the Adams method and outcomes achieved through ANN-based techniques.
3. It consistently provides high accuracy, typically within the range of 10^(-12) to 10^(-13), which underscores the effectiveness and reliability of the proposed ANN-BRM method.
4. ANN-BRM offers a natural and principled approach to combining prior information with data, all within a robust theoretical framework.

#### Future directions

In the future^41^, we will discuss stochastic solution of model^42^, fractional order drinking water model^43^, dynamics of food allergy^44^, delayed hepatitis B epidemic model^45^, stochastic cholera epidemic model^46^, and delayed stochastic HBV epidemic model^47^.

## Data Availability

All data generated or analyzed during this study are included in this published article.
